# Microbiological features of drowning-associated pneumonia: a systematic review and meta-analysis

**DOI:** 10.1186/s13613-024-01287-1

**Published:** 2024-04-20

**Authors:** Vladimir L. Cousin, Laure F. Pittet

**Affiliations:** 1 Intensive Care Unit, Department of Pediatric, Gynecology and Obstetrics, University Hospital of Geneva, University of Geneva, Rue Gabrielle-Perret-Gentil 4, 1206 Geneva, Switzerland; 2 Infectious Diseases, Immunology and Vaccinology Unit, Department of Pediatric, Gynecology and Obstetrics, University Hospital of Geneva, University of Geneva, Geneva, Switzerland

**Keywords:** Drowning associated pneumonia, Pneumonia, Intensive care unit, Antibiotic, Resistance

## Abstract

**Background:**

Drowning-associated pneumonia (DAP) is frequent in drowned patients, and possibly increases mortality. A better understanding of the microorganisms causing DAP could improve the adequacy of empirical antimicrobial therapy. We aimed to describe the pooled prevalence of DAP, the microorganisms involved, and the impact of DAP on drowned patients.

**Methods:**

Systematic review and meta-analysis of studies published between 01/2000 and 07/2023 reporting on DAP occurrence and microorganisms involved.

**Results:**

Of 309 unique articles screened, 6 were included, involving 688 patients. All were retrospective cohort studies, with a number of patients ranging from 37 to 270. Studies were conducted in Europe (France N = 3 and Netherland N = 1), United States of America (N = 1) and French West Indies (N = 1). Mortality ranged between 18 to 81%. The pooled prevalence of DAP was 39% (95%CI 29–48), similarly following freshwater (pooled prevalence 44%, 95%CI 36–52) or seawater drowning (pooled prevalence 42%, 95%CI 32–53). DAP did not significantly impact mortality (pooled odds ratio 1.43, 95%CI 0.56–3.67) but this estimation was based on two studies only. Respiratory samplings isolated 171 microorganisms, mostly Gram negative (98/171, 57%) and mainly *Aeromonas *sp. (20/171, 12%). Gram positive microorganisms represented 38/171 (22%) isolates, mainly *Staphylococcus aureus* (21/171, 12%)*.* Water salinity levels had a limited impact on the distribution of microorganisms, except for *Aeromonas* sp. who were exclusively found following freshwater drowning (19/106, 18%) and never following seawater drowning (0%) (p = 0.001). No studies reported multidrug-resistant organisms but nearly 30% of the isolated microorganisms were resistant to amoxicillin-clavulanate, the drug that was the most commonly prescribed empirically for DAP.

**Conclusions:**

DAP are commonly caused by Gram-negative bacteria, especially *Aeromonas* sp. which is exclusively isolated following freshwater drowning. Empirical antimicrobial therapy should consider covering them, noting than amoxicillin-clavulanate may be inadequate in about one-third of the cases. The impact of DAP on patients’ outcome is still unclear.

**Supplementary Information:**

The online version contains supplementary material available at 10.1186/s13613-024-01287-1.

## Introduction

Drowning is defined as a respiratory impairment following immersion or submersion of the airways in a liquid, typically water [1]. The ensuing hypoxemia and cardiac arrest carry a high mortality rate even with a brief period of immersion [1]. In survivors, aspiration of water in the alveoli causes surfactant dysfunction and washout, leading to diffuse alveolar damage and pulmonary edema. In 12% to 51% of the cases [2–4], survivors develop drowning-associated pneumonia (DAP), following the inhalation of contaminated water, endogenous flora or gastric content [1, 5]. DAP significantly impacts patient’s evolution, with prolonged mechanical ventilation and possibly higher mortality rate [2].

Limited data is available on microorganisms causing DAP; several factors can influence the microbial composition of contaminated water, including its chemical composition, geographic location and salinity level [6]. Ignoring the microorganisms causing DAP can adversely affect patients’ outcomes, through the administration of inappropriate empirical antimicrobial treatments. Indeed, a fair proportion of the microorganisms isolated in DAP are intrinsically resistant to the antimicrobials agents commonly recommended for community-acquired inhalation pneumonia. As suboptimal empirical antimicrobial therapy may lead to an unfavorable outcome, a better understanding of the causative microorganisms is needed [3].

The study aims to summarize the current knowledge on the ecology of microorganisms involved in DAP, and to assess the repercussion of DAP on patient outcomes.

## Methods

### Data sources and search strategy

Pubmed and EMBASE database were searched in August 2023 for relevant peer-reviewed articles, published in English or French, between January 2000 and July 2023, with no age restriction, in accordance with the PRISMA guidelines [7]. The following items were used for searches: (drowning associated pneumonia); (near-drowning associated pneumonia); (drowning AND pneumonia); (near-drowning AND pneumonia); (drowning AND microbiology). The references of all relevant publications were reviewed, and no further articles were identified.

One reviewer (VLC) screened the titles and abstracts to determine eligibility. Inclusion criteria were the following: studies reporting microbiological data on 10 humans or more who had survived drowning and later developed a DAP while being in intensive care units (ICU). Reviews and isolated case reports were excluded.

### Data collection

The following baseline data was extracted: year of publication; geographical setting; severity of drowning (i.e. occurrence of pre-admission cardiac arrest, requirement of mechanical ventilation, occurrence of acute respiratory distress syndrome [8], and admission Simplified Acute Physiology Score II (SAPS II) [9] and/or Sequential Organ Failure Assessment (SOFA) [10]); patients outcome (i.e. mortality, and duration of mechanical ventilation); water location (i.e. sea, lake, river, damp, pond, swimming pool, or miscellaneous) and salinity (i.e. seawater or freshwater).

Confirmed DAP was established by microbiology. The variables of interest for DAP included: numbers of respiratory samples; proportion of positive samples; type of microorganisms isolated; number of positive individual microorganisms isolated in respiratory samples; technic of respiratory sampling (i.e. broncho-alveolar lavage (BAL), protected specimen brush, tracheal aspirates, or sputum); antimicrobials used for empirical therapy; antimicrobial resistance in the microorganisms isolated.

### Statistics analysis

Descriptive statistics were used: continuous variables were reported as median (interquartile range IQR) and categorical variables as proportion (%). Microorganisms isolated from patients with seawater DAP were compared to those isolated from patients with freshwater DAP using a Fisher’s exact test. Stata v18 meta-analysis software pack was used to calculate the pooled prevalence of DAP (overall, following freshwater drowning and following seawater drowning) and to calculate the combined mortality odds ratio following DAP. Heterogeneity was assessed using the I^2^ statistics. High heterogeneity was defined as a I^2^ > 50%. In case of high heterogeneity, random-effect analyses were done and presented using forest plots. Stata v18 (StataCorp, College Station, TX, USA) was used for graphical and statistical analyses.

## Results

### Review and population

**Fig. 1:**
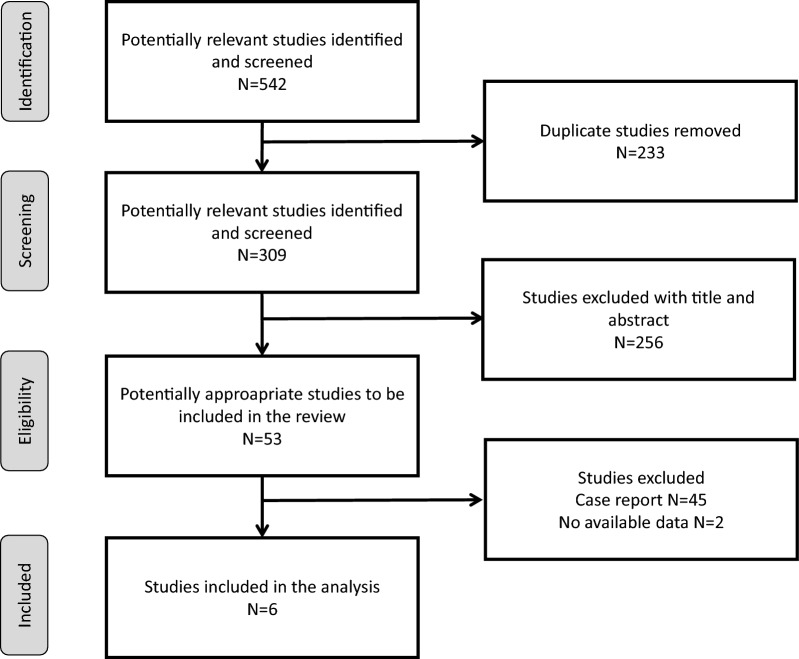
Preferred reporting items for systematic reviews and meta-analyses flow diagram

Of 309 unique articles, 6 studies were included, with a period of publication ranging from 2012 to 2023 (Fig. 1) [2, 3, 11–14]. Their main characteristics are detailed in Table 1. All were retrospective cohort studies, with a number of patients ranging from 37 to 270. Studies were conducted in Europe (France N = 3 and Netherland N = 1), United States of America (N = 1) and French West Indies (N = 1). Four studies included exclusively adult patients [2, 3, 11, 14], one included a mixed population of adult and pediatric patients [12] and the last one included only pediatric patients [13].

**Table 1:** Studies characteristics

	Tadié et al 2012	Assink-de Jong et al 2014	Cerland et al. 2017	Robert et al 2017	Moffett et al 2021	Reizine et al 2023
Country	France	Netherland	French West Indies	France	USA	France
Nb of patients	37	49	144	74	114	270
Age	37 (IQR 30–48)	ND	45 (IQR 19–60)	68 (IQR 51–77)	3.7 (IQR0.1–17.8)	68 (IQR 54–75)
Pre-ICU cardiac arrest	18 (49)	ND	61 (41)	30 (41)	89 (78)	103 (38)
Admission SAPS2	ND	ND	ND	45 (IQR 30–65)	ND	36 (IQR 26–83)
Admission SOFA	ND	ND	ND	ND	ND	3 (IQR 2–8)
Mechanical ventilation	ND	ND	64 (44)	51 (69)	53 (46.5)	114 (42)
ARDS	ND	ND	23 (16)	25 (34)	ND	102 (38)
Respiratory samples						
Nb	21 (57)	ND	ND	24 (33)	27 (24)	72 (27)
Nb positive	19/21 (90)	18/ND	22/ND	16/24 (67)	10/27 (37)	33/72 (46)
DAP						
Nb of DAP	19 (51)	18 (36)	35 (24)	36 (49)	ND	101 (37)
Nb of confirmed DAP	19/19 (100)	18/18 (100)	22/35 (63)	16/36 (44)	ND	56/101 (55)
Outcome						
Deceased	30 (81)	ND	45 (31)	19 (26)	21 (18.4)	55 (20.4)
Duration mechanical ventilation (days)	ND	ND	ND	1 (IQR 0–4)	3.5 (IQR 0.1–27)	3 (IQR 2–6)

ARDS acute respiratory distress syndrome; DAP drowning-associated pneumonia; ICU intensive care unit; Nb number of respiratory samples; ND non-documented; SAPS2 Simplified Acute Physiology Score II; SOFA Sequential Organ Failure Assessment; United States of America

A total of 688 patients were available for analysis. Location of drowning included sea (N = 393), swimming pool (N = 127), river (N = 55), pond (N = 30), bathtub (N = 19), other water source (N = 9), lake (N = 5) and swamp (N = 1). Location of drowning was not documented in 49 cases. As depicted in Table 1, a high proportion of patients presented pre-admission cardiac arrest, ranging from 38 to 78%, and the overall outcome was poor with mortality rate ranging between 18 and 81%.

### Drowning-associated pneumonia

**Fig. 2:**
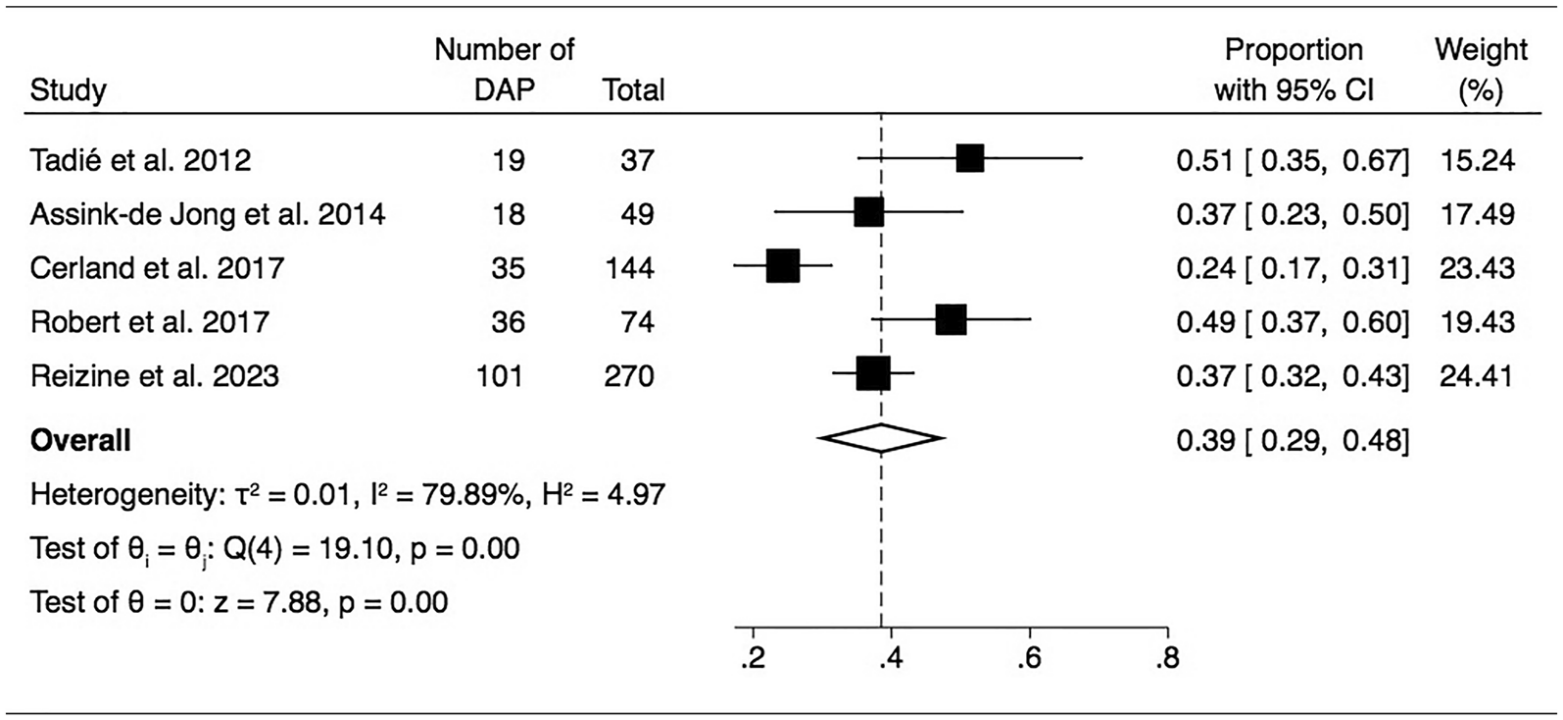
Forrest plot of included studies reporting prevalence of drowning associated pneumonia *DAP* drowning associated pneumonia

Five of the 6 studies reported the prevalence of DAP, ranging between 24 to 51%; the criteria they used to diagnose DAP are summarized in Table 2. The pooled prevalence of DAP was 39% (95% CI 29–48) (ι^2^ 0.01) (Figure 2) and was not influenced by the water salinity level, with similar pooled prevalence following freshwater DAP (44%, 95% CI 36–52) or seawater DAP (42%, 95% CI 32–53) (Additional file 1: Figure S1).

**Table 2:** DAP criteria used in reviewed studies

	Tadié et al 2012	Assink-de Jong et al 2014	Cerland et al 2017	Robert et al 2017	Reizine et al 2023
Fever (temperature cut-off)		x (38 °C)	x (38 °C)	x (38.5 °C)	x (38 °C)
Purulent secretion	x	x	x	x	x
Leucocytes count	x (< 5000/mm^3^ or > 12 000/mm^3^)	x (< 4000/mm^3^ or > 10 000/mm^3^)	x (< 4000/mm^3^ or > 10 000/mm^3^)	x (< 4000/mm^3^ or > 10 000/mm^3^)	x (< 4000/mm^3^ or > 10 000/mm^3^)
New infiltrate on a chest X-ray	x	x	x	x	x
Confirmed with a positive microorganism	x	x	x	x	x
Timing after admission	< 48 h	< 7 days^a^	< 48 h	< 48 h	< 48 h

^a^16/18 of DAP occurred in the first 48 h of ICU admission

Use of prophylactic antibiotics was not reported to be a routine procedure in any of the 6 studies. When DAP was suspected, various empirical antimicrobial therapies were used, predominately amoxicillin-clavulanate (Table 3). Only 3/6 studies evaluated the adequacy of the empirical antimicrobial therapy for the isolated microorganism, ranging from 50 to 89% [2, 3, 14] .

**Table 3:** Empiric antimicrobial therapy

	Tadié et al. 2012	Assink-de Jong et al. 2014	Cerland et al. 2017	Robert et al. 2017	Moffett et al. 2021	Reizine et al. 2023
Amoxicillin-clavulanate	17 (80)	0	78 (92)	34 (94)	1 (2)	69 (78)
Cefotaxime	0	0	5 (6)	0	11 (19)	0
Cefotaxime + metronidazole	0	0	2 (2)	0	0	8 (9)
Piperacillin-tazobactam	4 (20)	ND^a^	0	2 (6)	8 (14)	4 (4)
Ceftazidim	0	ND^a^	0	0	2 (4)	0
Other	0	0	0	0	35 (61)	8 (9)

^a^Assink-de-Jong reported using either piperacillin-tazobactam or ceftazidime but did not provide participant level details

*ND* non documented

**Fig. 3:**
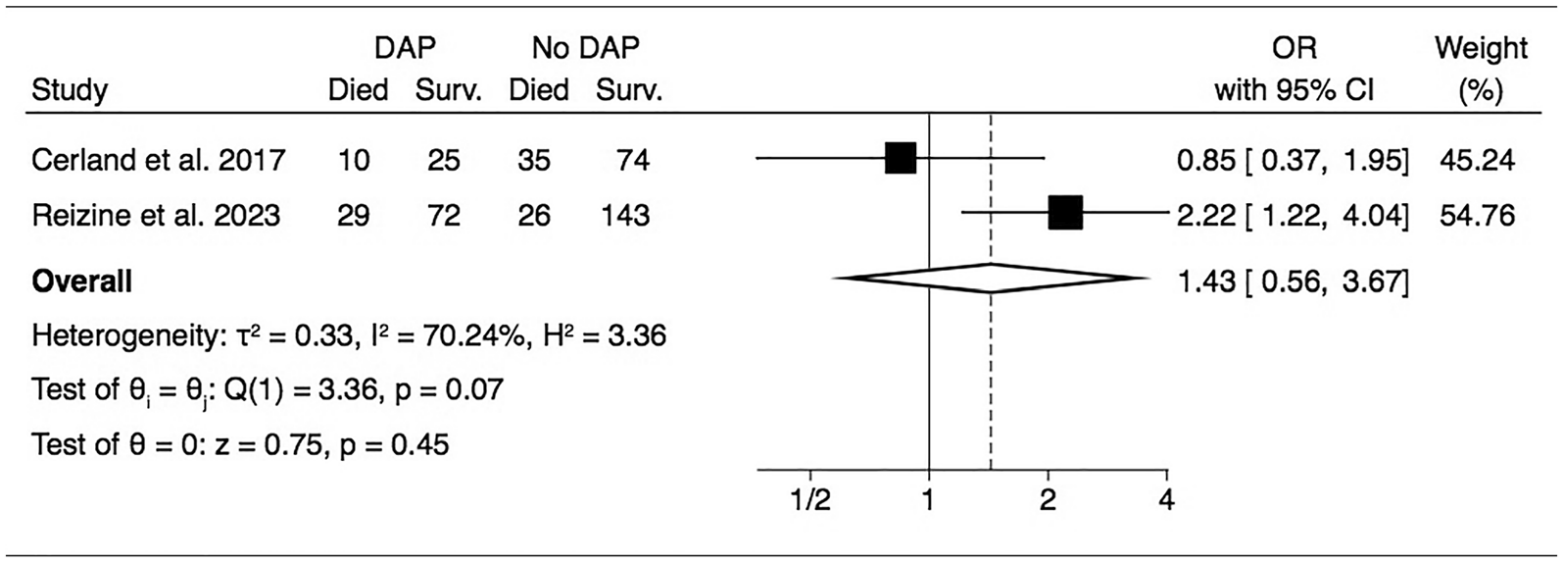
Forrest plot of included studies reporting impact of DAP on patients’ mortality rate *DAP* drowning associated pneumonia; *Surv* survivor

 The impact of DAP on patient’s outcome was reported inconsistently across studies, with only 2 studies reporting individual-level data enabling to estimate the impact of DAP on patient outcome [2, 12]. With a total of 414 patients and 136 DAP, the meta-analysis suggests a negative impact of DAP on survival, although not statistically significant (pooled odds ratio 1.43, 95% CI 0.56–3.67) (Fig. 3).

### Isolated microorganisms

A total of 171 microorganisms were isolated from 167 respiratory samples (including bronchoalveolar lavage (30%), protected specimen brush (9%), tracheal aspirates (35%), sputum (3%), and 24% not documented), as detailed in Table 4. Gram-negative were predominant (N = 98/171 (57%), primarily *Aeromonas *sp. (N = 20/171 (12%)), *Haemophilus influenzae* (N = 19/171 (11%)) and *Pseudomonas aeruginosa* (N = 12/171 (7%)). Gram positive followed with 38/171 (22%) isolates, mainly *Staphylococcus aureus* (N = 21/171 (12%)). Multiples germs were isolated in 10% of samples. Fungi were detected in a minority of samples with only 2/171 *Candida* sp. (1%) and 1/171 (0.5%) *Aspergillus* sp.

**Table 4:** Microorganisms isolated from respiratory samples

	Total ^a^ (N = 171)	Seawater (N = 43)	Freshwater (N = 106)	p value	Presumed susc AMC	Presumed susc CFT	Presumed susc PTZ
*Aeromonas* sp.	20 (11.7)	0	19 (17.9)	0.001	−	+	+
*Haemophilus influenzae*	19 (11.1)	7 (16.3)	12 (11.3)	0.4	+	+	+
*Pseudomonas aeruginosa*	12 (7.0)	5 (11.6)	6 (5.6)	0.3	−	−	+
*Enterobacter cloacae*	8 (4.7)	3 (6.9)	3 (2.8)	0.3	−	±	+
*Escherichia coli*	7 (4)	3 (6.9)	4 (3.8)	0.4	+	+	+
*Enterobacter aerogenes*	5 (2.9)	3 (6.9)	0	0.02	−	±	+
*Citrobacter koserii*	4 (2.3)	2 (4.6)	2 (1.9)	0.6	+	+	+
*Klebsielle* sp*.*	4 (2.3)	2 (4.6)	2 (1.9)	0.6	±	+	+
Gram negative, unspecified	4 (2.3)	0	4 (3.8)	0.3	?	?	?
*Morganella morganii*	2 (1.1)	0	1 (0.9)	1	−	±	+
*Moraxella catarrhalis*	2 (1.1)	0	2 (1.8)	1	+	+	+
*Acinetobacter* sp.	2 (1.1)	0	2 (1.9)	1	−	−	±
*Stenotrophomonas maltophilia*	2 (1.1)	0	2 (1.9)	1	−	−	±
*Citrobacter freundi*	1 (0.6)	0	1 (0.9)	1	−	±	+
*Klebsiella pneumoniae*	1 (0.6)	1 (2.3)	0	0.3	+	+	+
*Klebsiella oxytoca*	1 (0.6)	1 (2.3)	0	0.3	+	+	+
*Bacteroides *sp.	1 (0.6)	0	1 (0.9)	1	+	−	+
*Vibrio alginolyticus*	1 (0.6)	1 (2.3)	0	0.3	?	+	+
*Serratia marcescens*	1 (0.6)	0	1 (0.9)	1	−	±	+
*Flavobacterium odoratum*	1 (0.6)	0	1 (0.9)	1	?	?	?
*Staphylococcus aureus*	21 (12.3)	6 (13.9)	13 (12.2)	0.8	+	+	+
*Streptococcus pneumoniae*	13 (7.6)	5 (11.6)	8 (7.5)	0.4	+	+	+
*Streptococcus pyogenes*	3 (1.7)	0	3 (2.8)	0.6	+	+	+
*Bacillus cereus*	1 (0.6)	0	1 (0.9)	1	−	−	−
Flora, unspecified	35 (20.5)	4 (9.3)	18 (16.9)	0.3	?	?	?
Polymicrobial	18 (10.5)	8 (18.6)	10 (9.4)	0.2	±	±	±
*Aspergillus* sp.	1 (0.6)	0	1 (0.9)	1	^b^	^b^	^b^
*Candida* sp.	2 (1.1)	0	2 (1.9)	1	^b^	^b^	^b^

Susceptibility of microorganism is based on theorical susceptibility of wild-type colonies (Based on EUCAST). Importantly, local antimicrobial resistance could vary widely and antimicrobial therapy should be adjusted to local microbiological ecology AMC amoxicillin-clavulanate; CFT cefotaxime; PTZ piperacillin-tazobactam; Susc susceptibility

^a^Water salinity of 22 samplings could not be found

^b^non applicable

Microorganisms were compared according to the water salinity level (Table 4). The proportion of Gram positive and Gram negative was similar across both types of water. *Aeromonas *sp. was exclusively detected following freshwater drowning, with 19/106 (18%) of positive samples, and never following seawater drowning (0%) (p value 0.001). *Enterobacter *sp. were more frequently detected following seawater drowning (6/43) compared to freshwater drowning (3/106; p value 0.01). Fungi were exclusively isolated following freshwater drowning.

### Antimicrobial therapy resistance and adequacy

Studies did not report systematically on antibiotic resistance. Three studies reported on the proportion of microorganisms being resistant to amoxicillin-clavulanate: 31% in Robert et al*.* [3], 36.4% in Reizine et al*.* [2] and 31.6% in Tadié et al*.* [14]. One study reported the prevalence of cefotaxime resistance to be 12% [2]. No studies reported on the presence of multidrug-resistant microorganisms.

Studies did not consistently report on the inadequacy of antimicrobial therapy and its consequences; however, some interesting results were mentioned by two studies. Reizine et al*.* reported a mortality rate of 7/10 (70%) among the patients who received inadequate antimicrobial therapy, whereas the global mortality rate in that study was 20% [2]. In the publication from Tadié et al*.* the mortality rate was 2/6 among the patients who received inadequate antimicrobial therapy, whereas the global mortality rate in that study was 81% [14].

## Discussion

In this systematic review, we assessed the impact of DAP on nearly 700 patients admitted to ICU following drowning. A variety of microorganisms were isolated, irrespective of the water salinity level, apart from *Aeromonas* sp. and fungi that were exclusively isolated following freshwater drowning. As the empirical antibiotic therapy used was usually not targeting the isolated microorganisms, our findings highlight the importance of early bacterial samplings in drowned patients, as inadequate treatment is likely to impact the patients’ outcome.

Drowning represents one of the leading causes of accidents worldwide and carries a high mortality rate [15]. Patients surviving the initial drowning event are often admitted to ICU and are at risk of secondary respiratory complications such as DAP. Historically, DAP prevalence was reported to be between 11% and 54% [4, 16–18], in line with our updated estimate of 39%. Importantly, not all patients developed DAP, possibly owing to multiple factors, including: different microorganism load, varying immersive liquid chemical composition, occurrence of laryngospasm preventing aspiration, and the nature of drowning (i.e. primary or secondary to seizure, syncope, arrhythmia, or trauma) [1, 6].

Whether DAP occurrence increases the mortality rate is still unknown. In the past, the mortality rate in patients with DAP ranged between 26% and 60%, whilst recent studies report a mortality rate of approximately 28% [2, 4, 6, 12]. We found conflicting results in our review, with a non-significant trend for an impact of DAP on mortality rate. Cerland et al*.* reported similar mortality rates among patients with or without DAP, while Reizine et al*.* suggested a detrimental impact of DAP on patient outcome [2, 12]. DAP can lead to hypotension, hypoxemia and temperature instabilities, all recognized as factors worsening patient outcome following a cardiac arrest [19, 20]. Moreover, the intricated influence of inflammation triggered by DAP and the consequence of an inflamed lung may have on brain lesions might also play a significant role in affecting the patient outcome [20, 21]. However, other factors may have more impact on patient’s outcome, such as pre-admission cardiac arrests, or patients’ comorbidities. Similarly, a study showed that the reduction of early ventilator-associated pneumonia occurrences in post-cardiac arrest patients did not improve the mortality rate or the duration of mechanical ventilation [22].

We underscore the high prevalence of Gram-negative bacteria, both in freshwater and seawater, as historically described [4, 6]. The high incidence of *Enterobacter* sp. and other coliform bacteria could be explained by water fecal contamination [23]. Identification of those microorganisms strongly suggests the inhalation of contaminated water. In addition, a large number of samples suggest aspiration of oro-pharyngeal secretions (*Streptococcus pneumonia*, *Staphylococcus aureus*, *Haemophilus influenza*). Those results underline the role of aspiration, from both water or secretion, as a source for bacterial inoculum in DAP.

Importantly, *Aeromonas* sp*.* was the main germ isolated following freshwater drowning. This microorganism has several chromosomal beta-lactamases, which can impact DAP management trough reduced susceptibilities to antimicrobial agents, such as amoxicillin-clavulanate (only 16% susceptible isolates in a report), the most commonly used antimicrobial agent for empirical treatment [24–26]. However, most of *Aeromonas* sp. may remain susceptible to cefepime or piperacillin-tazobactam [25]. Despite its aquatic tropism, *Pseudomonas aeruginosa* was isolated in less than 10% of the samples. The density of *Pseudomonas* spp. colony in water is highly variable and may be very low in surface waters of natural water area, while contamination may be significant in recreational waters such as swimming pools [27]. Noticeably, fungal or anaerobic identification was rare. However, in special circumstances such as natural disasters, high incidence of *Aspergillus* sp. has been reported [28]. Considering those germs in these specific situations seems to be a practical approach to adopt [11].

The dilemma of whether empirical antimicrobial therapies are indicated at admission of drowned patient remains unresolved, but most guidelines discourage using them systematically [4, 29, 30]. A practical approach would be to restrain the use of such antimicrobial in drowned patients, with the exception of drowning occurring in highly contaminated environments (e.g. septic tank, manure pit) or in patients presenting severe lung lesions. As only a third of patients may develop a DAP, early respiratory sampling seems reasonable when DAP is suspected, as it has been shown to be effective to reduce antimicrobial prescription in patients with aspiration pneumonia and may help to guide antimicrobial therapy or help cease it [31]. Respiratory samplings will enable to rapidly identify the causative microorganisms and its antimicrobial susceptibilities. As the main isolated germs are Gram-negative, including *Aeromonas* spp. or *Pseudomonas* spp., close follow-up of antimicrobial susceptibilities is crucial as clinicians may encounter resistant microorganisms causing DAP. Antimicrobial resistance in the environment could be frequent through acquiring and sharing antibiotic resistance genes, in addition to natural resistance [32]. When antibiotic treatment cannot be delayed, piperacillin-tazobactam or a 4th generation cephalosporin could be suggested as first-line treatment, since inadequate antimicrobial therapy seems to carry a high risk of adverse outcome, as mentioned in the reviewed studies [2, 3, 11, 14]. Importantly, antimicrobial therapy should always be tailored to local microbiological ecology and de-escalation performed as soon as possible.

It is important to note that diagnosis of DAP remains difficult as numerous criteria used for its definition can be confounded by concurring events, similarly to ventilator associated pneumonia [33]. The use of controlled temperatures after a cardiac arrest may mask any sign of hypo or hyperthermia linked to an infection, as illustrated by Reizine et al. who reported a median body temperature of 38.1 °C (IQR 35.6–38.7) at DAP diagnosis [2]. Interpreting radiological findings can be challenging in presence of lung damage and difficult to differentiate DAP from cardiogenic pulmonary edema, atelectasis and non-infective acute lung injury related to submersion [5]. In addition, inflammatory markers may be less useful following cardiac arrest as they will be deranged by the ischemia–reperfusion syndrome [34]. All these considerations highlight the importance of maintaining a low threshold for respiratory samplings in drowned patients, as it serves as a crucial criterion to initiating treatment and will be paramount to adjust the antimicrobial therapy.

### Limitations

Our study has several limitations, including publication bias. All included studies were retrospectives; they differed in their methodology and their population in terms of proportion of pre-hospital cardiac arrests, severity score at admission, and whether they included drowned and/or nearly-drowned patients. The results may not apply to all geographic areas, especially tropical and/or warm temperature waters (only 1/6 studies take place in a tropical area, the one from the French West Indies). Moreover, meta-analyses on the impact of DAP on patients outcome could only be performed by 2 studies. Not all patients with DAP had a respiratory sampling done, and the microorganisms identified are not necessarily the causative agents of DAP. Finally, as mentioned above, diagnosis of pneumonia remains difficult in this population and some patients may have drowning-induced pulmonary damage misdiagnosed as DAP.

## Conclusion

This study provides important information on DAP ecology, emphasizing the predominant role of Gram-negative bacteria and *Aeromonas* sp. who are commonly resistant to the antimicrobial frequently used empirically. As amoxicillin-clavulanate does not cover the microorganisms commonly isolated, piperacillin-tazobactam or a 4th generation cephalosporin could be more suitable for empirical treatment. When empirical therapy is required, respiratory sampling should be performed, and potential resistance should be investigated. Future studies are needed to investigate the impact of DAP on patient outcome and the role of an early antimicrobial therapy in drowned patients.

### Supplementary Information

**Additional file 1:** **Table. S1.** Forrest plot of included studies reporting prevalence of drowning associated pneumonia, depending on the salinity of the water DAP drowning associated pneumonia

## Data Availability

Data are available on reasonable request.

## Abbreviations

DAP: Drowning-associated pneumonia
ICU: Intensive care unit
IQR: Interquartile range
SAPS II: Simplified acute physiology score II
SOFA: Sequential organ failure assessment
