# Supplementary material for: Microbiological features of drowning-associated pneumonia: a systematic review and meta-analysis
Source: Ann Intensive Care. 2024 Apr 20;14:61. doi: 10.1186/s13613-024-01287-1 (PMC11031557; doi:10.1186/s13613-024-01287-1)
Supplement: Supplementary file 1 — Additional file 1: Table. S1. Forrest plot of included studies reporting prevalence of drowning associated pneumonia, depending on the salinity of the water DAP drowning associated pneumonia [file 13613_2024_1287_MOESM1_ESM.docx]

**Supplementary figure 1**

**Forrest plot of included studies reporting prevalence of drowning associated pneumonia, depending on the salinity of the water**


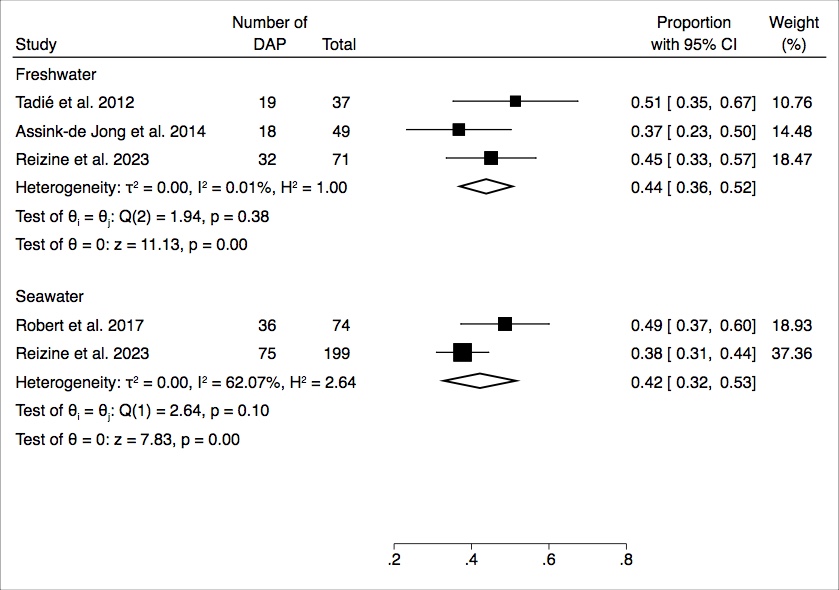


DAP drowning associated pneumonia
